# Functional Connectivity Alterations in Children with Spastic and Dyskinetic Cerebral Palsy

**DOI:** 10.1155/2018/7058953

**Published:** 2018-08-15

**Authors:** Yun Qin, Yanan Li, Bo Sun, Hui He, Rui Peng, Tao Zhang, Jianfu Li, Cheng Luo, Chengyan Sun, Dezhong Yao

**Affiliations:** ^1^The Clinical Hospital of Chengdu Brain Science Institute, MOE Key Lab for Neuroinformation, High-Field Magnetic Resonance Brain Imaging Key Laboratory of Sichuan Province, University of Electronic Science and Technology of China, Chengdu 610054, China; ^2^Sichuan Rehabilitation Hospital, Chengdu, China

## Abstract

Cerebral palsy (CP) has long been investigated to be associated with a range of motor and cognitive dysfunction. As the two most common CP subtypes, spastic cerebral palsy (SCP) and dyskinetic cerebral palsy (DCP) may share common and distinct elements in their pathophysiology. However, the common and distinct dysfunctional characteristics between SCP and DCP on the brain network level are less known. This study aims to detect the alteration of brain functional connectivity in children with SCP and DCP based on resting-state functional MRI (fMRI). Resting-state networks (RSNs) were established based on the independent component analysis (ICA), and the functional network connectivity (FNC) was performed on the fMRI data from 16 DCP, 18 bilateral SCP, and 18 healthy children. Compared with healthy controls, altered functional connectivity within the cerebellum network, sensorimotor network (SMN), left frontoparietal network (LFPN), and salience network (SN) were found in DCP and SCP groups. Furthermore, the disconnections of the FNC consistently focused on the visual pathway; covariance of the default mode network (DMN) with other networks was observed both in DCP and SCP groups, while the DCP group had a distinct connectivity abnormality in motor pathway and self-referential processing-related connections. Correlations between the functional disconnection and the motor-related clinical measurement in children with CP were also found. These findings indicate functional connectivity impairment and altered integration widely exist in children with CP, suggesting that the abnormal functional connectivity is a pathophysiological mechanism of motor and cognitive dysfunction of CP.

## 1. Introduction

Cerebral palsy (CP) is the most common cause of physical disability in early childhood, occurring at a rate of around 2 per 1000 live births [1, 2]. CP encompasses a range of motor and postural disorders resulting from nonprogressive injury during the prenatal or infant development, causing serious activity limitation often accompanied by various degrees of sensation and cognition impairments [3, 4]. Spastic cerebral palsy (SCP) is the most common CP subtype, which is usually presented with bilateral spastic or hemiplegic disability with increased muscle tone, hyperreflexia, and persistence of primitive reflexes [5]. Dyskinetic CP (DCP) is the second common subtype, affecting 15%–20% of children with CP [6]. The new definition of dyskinetic CP includes dystonic and choreoathetoid CP, which is characterized by abnormal movement or posture, with involuntary, recurring, uncontrolled, and occasionally stereotyped movements [7, 8]. Besides motor function impairment, both SCP and DCP subtypes tend to be associated with visual and auditory impairment and cognitive impairments such as intellectual and learning disability [1, 7]. Furthermore, evidence points to abnormal sensorimotor reorganization, attention, and executive function deficits, as well as visual-perceptual impairments in children with DCP and SCP [9–11].

The wide array of dysfunction in CP is due to the heterogeneous nature of the underlying cerebral lesions. Depending on the location, extent, and timing of the insult, clinical symptoms vary largely [12]. Brain maldevelopment, white matter (WM) lesions, basal ganglia lesions, and cortical/subcortical lesions [13, 14] are the most common pathological findings in CP. Bilateral spastic CP (diplegia and tetraplegia) is foremost associated with white matter injury especially periventricular leukomalacia (PVL), in particular in children born preterm or prolonged hypoxic-ischemic events [15, 16]. Some MR studies have showed that brain injury pattern of DCP was associated with basal ganglia and thalamic injury following profound hypoxic insults and commonly seen in term infants [17, 18]. Cortical injury and WM involvement have also been reported in DCP cases [13]. Nevertheless, the heterogeneity among neuroimaging studies makes it ambiguous to systematically understand the relationship between the dysfunction and the brain state in children with CP.

Previous studies have widely applied diffusion tensor imaging to focus on the microstructural damage of white matter fiber tract of CP [19, 20]. Inverse correlation was found between the quantitative indicators of sensory-motor tracts and gross motor clinical grade in CP group [21]. In addition, some voxel-based morphometry studies have investigated that the gray matter abnormality in CP was associated with clinical features [22–24]. However, the common and distinguished brain dysfunctional characteristics associated with clinical features between DCP and SCP are relatively less explored.

Functional magnetic resonance imaging (fMRI) has been effectively used in children with CP to map the regional motor and cognitive processing [25–27]. Applications of fMRI in children with CP have mainly focused on the somatosensory, motor, and language tasks which are directly associated with the sensorimotor dysfunction in CP [28, 29]. Other cognitive impairments widely existing in children with CP, such as visual-perceptual, attention, and executive function [9–11, 30], are less explored because performing relative tasks during fMRI scanning may be difficult for children with CP. In contrast, the resting-state fMRI scanning is simple and easy to execute for these children [31]. Functional connectivity, referring to an analysis for identifying spatial patterns of coherent BOLD activity in distributed brain regions, has been used to detect the abnormal pathways (e.g., somatosensory, motor, and thalamocortical pathway) in children with CP using resting-state fMRI data [22, 32, 33]. For defining distinct modes of long-distance interactions, independent component analysis (ICA) has been a popular method to generate resting-state networks (RSNs) [34, 35], and resting-state functional network connectivity (FNC) [36] can be used to represent the temporal correlation among these RSNs. Therefore, investigations of the RSNs and FNC may provide more information to advance the understanding of the underlying physiopathology mechanisms in children with SCP and DCP. We hypothesized that common and distinct functional connectivity patterns may exist in SCP and DCP.

To test our hypothesis, 16 children with DCP, 18 children with SCP, and 18 healthy controls were included to explore the resting-state functional connectivity within and between RSNs. Group-level independent component analysis [37, 38] was used to extract the RSNs, and FNC analysis was performed. Group comparisons were then conducted for RSNs and FNC. The relationship between functional disconnection and motor-related clinical measurement in CP was also examined.

## 2. Material and Methods

### 2.1. Participants

A total of 43 children with CP were recruited from Sichuan Rehabilitation Hospital: 22 DCP (10 female, mean age: 9 years, age range: 3–18 years) and 21 SCP (10 female, mean age: 9.2 years, age range: 3–16 years) were involved. The diagnosis for the two subtypes of CP was made through standardized assessment by neurologists based on clinical features and MRI scanning [7]. All children with SCP were diplegic, and all children with DCP were dystonic. The inclusion criteria for the study were as follows: (1) a diagnosis of DCP and SCP with predominant spastic diplegic or dyskinetic features, (2) age under 18 years old, and (3) no history of trauma or brain operation. Children with serious brain loss or lesions (lesion size > 1 cm^3^, including 4 DCP children and 2 SCP children) were excluded based on MRI brain image. No child was on medication. 20 healthy children without history of neurological disorder or brain injury (7 female, mean age: 9.3, age range: 5–12) were included in the healthy control group (HC). Parents of all participants gave written informed consent in accordance with the Declaration of Helsinki. This study was performed according to the guidelines approved by the Ethics Committee of the University of Electronic Science and Technology of China (UESTC).

### 2.2. Clinical Measurement

Motor function was assessed for the DCP and SCP groups by the Gross Motor Function Classification System (GMFCS) [39]. GMFCS scores range from level I, which indicates children with no disability for community mobility, to level V, which includes children who are totally dependent on assistance for mobility. Everyday activities of daily living (ADL) [40] were also evaluated by the Assessment of Motor and Process Skills (AMPS) to access the self-care ability during everyday life. Both GMFCS and ADL evaluations were conducted by physical therapists in the Rehabilitation Assessment Department, Sichuan Rehabilitation Hospital.

### 2.3. Imaging Data Acquisition

Images were acquired on a 3 T MRI scanner (GE Discovery MR750) at the MRI Research Center of UESTC. Children with CP underwent MRI scanning under monitored sedation induced by midazolam (0.1 mg/kg) and propofol (1-2 mg/kg), which is approved by the Department of Anaesthesia of the Sichuan Rehabilitation Hospital, and written informed consent was obtained from participants' parents. The HC children were instructed simply to keep their eyes closed and remain still without sedation. The same scanning protocol was used for both CP and HC children as follows. High-resolution T1-weighted images were acquired using a 3-dimensional fast spoiled gradient-echo (T1-3D FSPGR) sequence (repetition time (TR) = 5.956 ms, echo time (TE) = 1.964 ms, flip angle (FA) = 9°, matrix = 256 × 256, field of view (FOV) = 25.6 × 25.6 cm^2^, slice thickness = 1 mm, no gap, 152 slices). T2 images were also acquired using OAx T2 fluid-attenuated inversion recovery (OAx T2 FLAIR) (TR = 8400 ms, TE = 150 ms, FA = 111°, matrix = 256 × 256, FOV = 25.6 × 25.6 cm^2^, slice thickness = 4 mm). Resting-state functional MRI data were acquired using gradient-echo echo-planar imaging sequences (TR = 2000 ms, TE = 30 ms, FA = 90°, matrix = 64 × 64, FOV = 24 × 24 cm^2^, slice thickness/gap = 4 mm/0.4 mm), with an eight channel-phased array head coil. A total of 255 volumes were collected from each participant.

### 2.4. fMRI Preprocessing

Data preprocessing was performed using SPM8 (statistical parametric mapping, http://www.fil.ion.ucl.ac.uk/spm/). The first ten volumes were discarded for the magnetization equilibrium from all fMRI scans. Then, the remaining images were slice-timing corrected and realigned (motion-corrected). The transition and rotation parameters were checked, and given the relatively large head motion of the children or teenagers during fMRI scanning, we increased the motion threshold to 3 mm for head movement and 3°for head rotation as the exclusion standard just as other studies did with adolescent participants [41–43]. Group comparison was performed for the individual mean framewise displacement (FD) calculated by averaging the relative displacement from every time point for each subject [44], and no difference was found between the groups (one-way ANOVA, *P* = 0.2439). The formula to calculate the FD is

(1) $$\begin{array}{c} FD=\frac{1}{M-1}\overset{M}{\underset{i=2}{\sum }}\sqrt{{\left|\Delta t_{x_{i}}\right|}^{2}+{\left|\Delta t_{y_{i}}\right|}^{2}+{\left|\Delta t_{z_{i}}\right|}^{2}+{\left|\Delta d_{x_{i}}\right|}^{2}+{\left|\Delta d_{y_{i}}\right|}^{2}+{\left|\Delta d_{z_{i}}\right|}^{2}}, \end{array}$$

where *M* is the length of the time courses; *x*_*i*_, *y*_*i*_, and *z*_*i*_ are translations/rotations at the *i*th time point in the *x*, *y*, and *z* directions, respectively; Δ*t* represents the framewise displacement translation; Δ*d* represents the framewise displacement rotation; and Δ*d*_*x*_*i*__ = *x*_*i*_ − *x*_*i*−1_, similar for Δ*d*_*yi*_, Δ*d*_*zi*_, Δ*t*_*xi*_, Δ*t*_*yi*_, and Δ*t*_*zi*_.

Spatial normalization was performed using the T1-based transformation. The individual T1 images were coregistered to the functional images and then segmented and normalized to the Montreal Neurologic Institute (MNI) space by a 12-parameter nonlinear transformation. Additionally, we used a cost-function modification to exclude the lesion area avoiding bias during spatial normalization [45]. The process has been implemented in SPM8 and adopted in other brain imaging studies with lesions in our lab [46]. The transformation parameters were applied to functional images. Then, functional data were resampled to 3 × 3 × 3 mm^3^ voxels after spatial normalization. Moreover, the images were spatially smoothed through convolution with a 6 mm full-width half-maximum (FWHM) Gaussian kernel.

### 2.5. Lesion Mapping

In this study, we constructed a lesion overlap image of CP. First, a radiologist marked the gray matter lesions on individual 3D T1 images. The lesions in this study mainly covered some voxels in the occipital region for one child and basal ganglia region for four children. Then, the union of all individual lesions was used to construct a group lesion mask after the spatial normalization process. At last, a specific group mask was generated from the gray matter template excluding the patients' group lesion mask for the next ICA analysis.

### 2.6. Independent Component Analysis

As a data-driven statistical analysis technique, ICA processing yields independent components (ICs) which represent a group of brain regions with a unique pattern of synchronized neural activity. Components with special spatial pattern can be selected as resting-state networks. For all CP children and healthy controls, group ICA analysis was performed to decompose the data into ICs using GIFT software [38] (version 2.0e; http://mialab.mrn.org/software/gift/). The specific group mask excluding the CP lesion mask was used in the group ICA. Principal component analysis (PCA) was adopted for the reduction of data dimensionality. The number of ICs was determined according to the minimum description length (MDL) [47]. In ICASSO (http://research.ics.tkk.fi/ica/icasso), the infomax algorithm was repeated 20 times as an independent component estimation. Then, the dual-regression (DR) approach was used in the back reconstruction step to back reconstruct the individual participant components [48]. Thus, IC time courses and spatial maps were acquired for each participant, and the participant-specific maps were converted to *Z*-score. In addition, for the validation of the selection of the separated ICs, we implemented the ICA using different independent component numbers (model order) and severally conducted the following analysis (i.e., within RSN analysis and FNC analysis) for seven times.

### 2.7. Within RSN Analysis

Among the 39 components resulting from ICA, 14 components were selected as nonartifactual RSNs through visual inspection in accordance with previously published results [49–52]. For each of the RSNs, *Z*-maps in each group were firstly gathered using the one-sample *t*-test for revealing group main effects, and the resulting statistical map was thresholded at *P* < 0.05 using the false discovery rate (FDR) correction. Then, group comparison was conducted for the *Z*-maps of the RSN using one-way ANOVA restricted to the voxels within a union mask, which was defined by the one-sample *t*-test results in three groups. Between-group effects were thresholded at *P* < 0.05 with voxel-wise FDR correction, and the minimum cluster size was 25 voxels. Regions (3^∗^3^∗^3 voxels) with high significant difference in ANOVA were chosen from each RSN and used in the post hoc analysis. During the one-way ANOVA and post hoc analysis, age and sex, as well as head-motion variables, were treated as unconcerned covariates.

### 2.8. FNC Analysis between RSNs

After ICA, the individual-level time courses of the identified RSNs were derived from the spatial-temporal dual regression. In order to investigate the relationship between time courses of different RSNs, FNC analysis was performed. First, temporal band-pass filter (band pass 0.01–0.1 Hz) was used to reduce the effects of low-frequency drift and high-frequency physiological noise on the time courses. Then, correlations were computed between the time courses of any two of the RSNs for each participant. Thus, individual potential internetwork connections (91 connections) were generated. After Fisher *Z*-translation of the correlation coefficients, one-sample *t*-test (*P* = 0.05, corrected by FDR) was applied to examine the significant temporal interactions between any two RSNs in DCP, SCP, and HC groups, respectively. To better understand the group difference of FNC, one-way ANOVA was performed for all potential connections between RSNs, with the significance cutoff *P* < 0.05 corrected by FDR to control for multiple comparisons. Then, the post hoc comparison (*P* < 0.01) was used to the connections with statistical significance in the ANOVA between groups. During the one-way ANOVA and post hoc analysis, age and sex, as well as head-motion variables, were treated as unconcerned covariates.

### 2.9. Correlations with Clinical Measurement

Correlations were calculated between the functional connectivity in RSNs/FNC and the clinical measurements of CP. For each RSN, the regions (3∗3∗3 voxels) with a peak *F* value in the ANOVA were selected as regions of interest (ROIs), and the coordinates of the ROIs were extracted. Then, the mean *Z*-scores within the ROI was used for the following correlation calculation. In addition, the coefficients of FNC with high significance in ANOVA were also used to calculate the correlation with the GMFCS and ADL scores.

### 2.10. Statistical Analyses

Before statistical analyses, normality of the data distribution was tested using the Lilliefors test, including the age, ADL scores, *Z*-map of RSNs for each voxel, and FNC connections. Then, to investigate the group difference for demographic and clinical data, chi-square test was applied to the categorical data including gender and GMFCS scores; one-way ANOVA and two-sample *t*-test were used for age and ADL scores, respectively. For RSN and FNC analysis, group comparison among the three groups was performed using one-way ANOVA. Significant differences revealed by ANOVA (*P* < 0.05, corrected by FDR) were further analyzed for multiple comparisons using Tukey's post hoc test. In addition, relationship between functional connectivity and clinical scores was examined using Pearson correlation for ADL and Spearman correlation for GMFCS, with statistical significance level *P* < 0.05 corrected by FDR. The voxel-level statistical analysis of RSNs was conducted using SPM8 (statistical parametric mapping), and the other statistical analysis, including the FNC group comparison and the correlation, was conducted using MATLAB functions (MATLAB 2015).

## 3. Results

### 3.1. Participants and Clinical and Radiological Findings

After the fMRI data head-motion checking, the final cohort in this study consisted of 16 DCP, 18 SCP, and 18 HC children. No significant difference was found for age and gender among the three groups (one-way ANOVA for age, *P* = 0.818; chi-square test for gender, *P* = 0.907). Comparing to SCP, DCP showed higher GMFCS (chi-square test, *P* = 0.003) and lower ADL (two-sample *t*-test, *P* < 0.001), indicating more severe motor and daily activity disability in the DCP group. Cerebral abnormalities were evaluated by two radiologists according to the high-resolution T1 (3D FSPGR) and T2 (OAx T2 FLAIR) images. Among 36 children, 5 children have gray matter lesions involving 1 child with small occipital lesion and 4 children with basal ganglia abnormality, mainly located in the bilateral putamen. 21 children have predominant white mater lesion types consisting of periventricular leukomalacia, ventricular enlargement, and other local white matter lesions. Demographic and clinical data of the sample were shown in Table 1.

### 3.2. RSN Identifications

**Figure 1:**
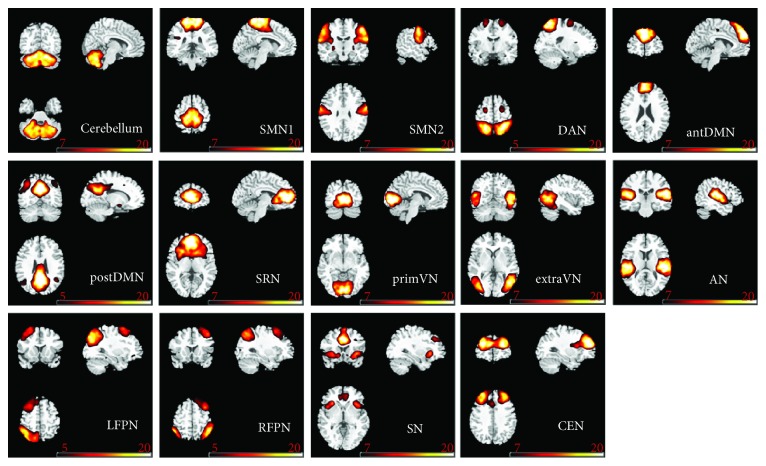
The spatial patterns of 14 RSNs identified according to the group ICA in all children. SMN: sensorimotor network; DAN: dorsal attention network; antDMN: the anterior part of default mode network; postDMN: the posterior part of default mode network; SRN: the self-referential network; primVN: the primary visual network; extraVN: the extrastriate visual network; AN: the auditory network; LFPN: the left lateral frontoparietal network; RFPN: the right lateral frontoparietal network; SN: the salience network; CEN: the central executive network.

Fourteen components were selected as the resting-state relevant networks from the group ICA in accordance with the previously published results [35, 51]. No clusters in each RSN fell within the lesion of any of the children. The spatial maps of the 14 RSNs are illustrated in Figure 1. These networks are labeled as follows: *cerebellum*: the spatial patterns primarily encompassed the cerebellum posterior lobe and declive; *SMN1*: sensorimotor network included the paracentral lobule, the supplementary motor area, and the pre- and postcentral gyrus; *SMN2*: sensorimotor network focused at the bilateral primary somatosensory cortex, including pre- and postcentral gyrus areas; *DAN*: dorsal attention network mainly included the bilateral intraparietal sulcus, frontal eye field, and middle temporal lobe; *antDMN*: the anterior part of default mode network (DMN) (antDMN) included the superior frontal gyrus and middle frontal gyrus; *postDMN*: the posterior part of DMN involved the posterior cingulate cortex (PCC), precuneus, and bilateral angular gyrus; *SRN*: the self-referential network mainly included the anterior cingulate and bilateral medial-ventral prefrontal cortex; *primVN*: the primary visual network showed the spatial patterns consisting of the cuneus, calcarine, and lateral lingual gyrus; *extraVN*: the extrastriate visual network encompassed the bilateral fusiform gyrus, middle temporal, and middle occipital areas; *AN*: the auditory network primarily encompassed middle temporal gyrus and superior temporal gyrus corresponding to the auditory system; *LFPN*: the left lateral frontoparietal network involved the left middle frontal gyrus, inferior parietal lobule, superior parietal lobule, and angular gyrus; *RFPN*: the right lateral frontoparietal network showed the similar spatial patterns with LFPN. LFPN and RFPN were the only maps strongly lateralized and left-right mirrors of each other; *SN*: the salience network showed spatial patterns mainly consisting of dorsal anterior cingulate (dACC) and orbital frontoinsular cortices, as well as part of prefrontal areas; *CEN*: the central executive network showed spatial patterns comprising the superior and middle frontal cortices, anterior cingulate, and paracingulate gyri. The group-level spatial maps of 14 RSNs for HC, SCP, and DCP were shown in Supplementary Material Figure S1.

### 3.3. Group Comparisons of Functional Connectivity within RSNs

**Figure 2:**
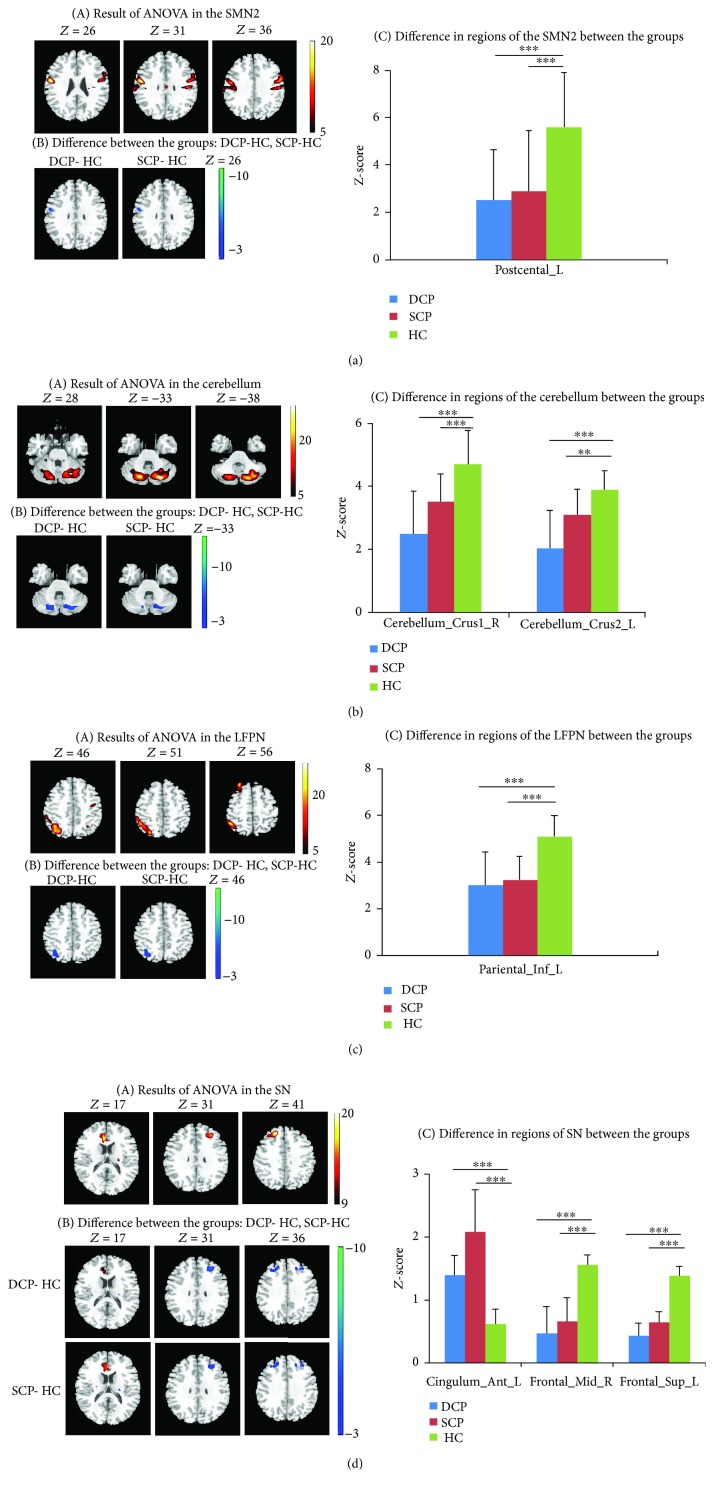
(a–d) Group comparison within four RSNs (SMN2, cerebellum, LFPN, and SN). (a) Significant difference was found in RSNs among the three groups. This result was achieved by performing one-way ANOVA on the maps of the three groups, with a threshold of corrected *P* < 0.05. (b) Differences were obtained between the DCP and HC group, as well as between the SCP and HC group, by performing post hoc test on the RSN maps (*P* < 0.001). (c) The bar maps present the between-group differences in the regions showing significant group difference. In the bar maps, ^∗∗^ *P* < 0.01, ^∗∗∗^ *P* < 0.001.

Group comparison of functional connectivity within RSNs was performed using one-way ANOVA. Significant difference among the DCP, SCP, and HC groups was found within four RSNs (*P* < 0.05, corrected by FDR), including the cerebellum network, SMN2, LFPN, and SN (Table 2). Post hoc analysis between the groups was performed within the regions where significant difference was observed in the three groups (Table 3, Table 4).  Figure 2 showed differences between the groups. Compared with HC, both DCP and SCP illustrated decreased functional connectivity within the cerebellum network, SMN2, and LFPN. Moreover, the SN revealed not only the reduced functional connectivity in middle frontal and superior frontal gyrus but also the increased functional connectivity in the anterior cingulum (ACC) in CP. No significant difference was found for functional connectivity within RSNs between the DCP and SCP group. In addition, according to the validation analysis using different independent component numbers in ICA processing, the results of functional connectivity within RSNs were mostly repeated in calculations seven times (details shown in Supplementary Material Figure S2).

### 3.4. FNC Analysis between Groups

**Figure 3:**
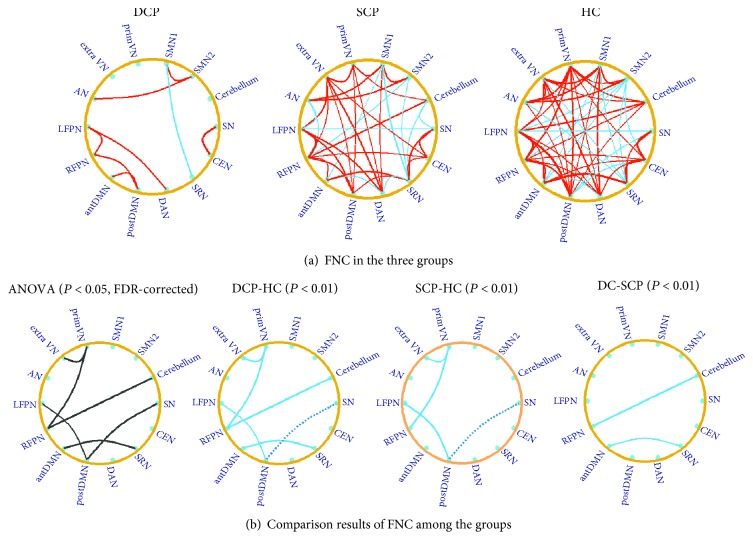
(a) The functional network connectivity in the DCP, SCP, and HC group. The red line represents the connection with positive correlation, and the blue line represents the connection with negative correlation. The results were obtained by the one-sample *t*-test with a threshold of corrected *P* < 0.05. (b) The comparison results of the functional network connectivity between the groups. The black line in the ANOVA result represents the altered connection among the three groups. Differences of functional network connectivity between DCP and HC and between SCP and HC, as well as between DCP and SCP, were observed. The blue solid line represents the connection with decreased positive FNC in CP children; and the blue dotted line represents the connection with decreased negative FNC in CP children.

For FNC analysis, significant internetwork connections were found for each group based on the one-sample *t*-test (*P* < 0.05, FDR-corrected) (Figure 3(a)). A large number of positive associations were detected between RSNs, and a small number of negative connectivity existed. Then, one-way ANOVA showed significant difference among the groups, and six connections were found to be significantly altered (*P* < 0.05, FDR-corrected), including the primVN-extraVN connection, primVN-RFPN connection, RFPN-cerebellum connection, antDMN-SRN connection, postDMN-LFPN connection, and postDMN-SN connection. Moreover, in the DCP group, all of the six connections were impaired (correlation coefficient approaching zero) compared with HC, while in the SCP group, only four out of the six disconnections existed. Comparing to SCP, DCP gave rise to more serious deficiency in the RFPN-cerebellum connection and antDMN-SRN connection. The altered FNCs were mostly involved in the validation analysis using different IC numbers (details shown in Supplementary Material Figure S3).

### 3.5. Relationship between Functional Connectivity and Clinical Scores

**Figure 4:**
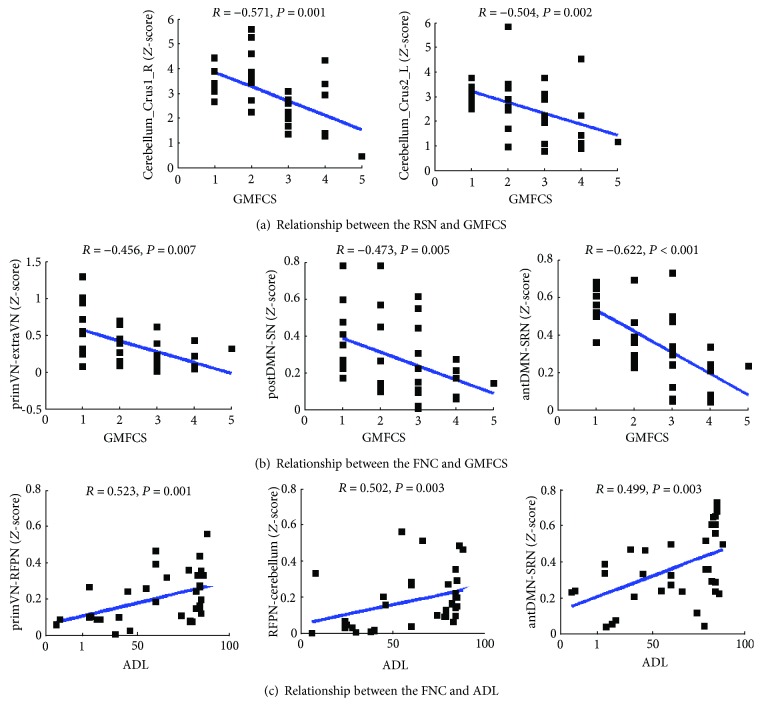
(a) Relationship between cerebellum network and GMFCS scores. (b) Relationship between primVN-extraVN, postDMN-SN, antDMN-SRN connection, and GMFCS scores. (c) Relationship between primVN-RFPN, RFPN-cerebellum, antDMN-SRN connection, and ADL scores.

Comparisons for clinical scores between the DCP and SCP group showed that GMFCS scores in the DCP group were significantly higher than those in the SCP group, and ADL scores in the DCP group were significantly lower than those in the SCP group. Correlations were performed between the mean *Z*-scores of seven ROIs in the four RSNs (Figure 2) and clinical scores, and significant negative correlations were found between the cerebellum crus and GMFCS (Figure 4(a)). Then, correlations were performed between the FNC coefficients (6 connections) and the clinical measurement in the CP group. Three out of the six connections, including primVN-extraVN and postDMN-SN, as well as antDMN-SRN, were found having negative correlations with GMFCS. Moreover, three connections including primVN-RFPN, RFPN-cerebellum, and antDMN-SRN were found having significant positive correlations with ADL scores (Figures 4(b)–4(c)). Besides the correlations in the whole group, relationship between functional connectivity and clinical scores was also investigated in the subgroups (Supplementary Material Figure S4).

## 4. Discussion

In this study, we investigated the functional connectivity intra- and inter-RSNs in CP. Compared with HC, altered functional connectivity was found within the cerebellum, SMN2, LFPN, and SN networks for both the SCP and DCP groups. For FNC analysis, four functional disconnection inter-RSNs were observed in SCP, while six functional disconnection inter-RSNs were observed in DCP. The DCP and SCP groups showed different levels of aberrant connectivity. Furthermore, correlations between the functional disconnection and GMFCS/ADL scores were found. These findings indicate functional connectivity impairment, and altered integration widely exists in children with CP, and exploring the common and distinct functional connectivity patterns may contribute to our understanding of the neuropathophysiological mechanism of different CP subtypes.

Functional connectivity analysis alteration within RSNs may elucidate the abnormal intrinsic interaction in a certain spatial pattern [34, 53]. In this study, decreased functional connectivity was showed within two motor-related networks, that is, the cerebellum and SMN2 in children with CP. The role of the cerebellum has been well known to be involved in both motor learning and cognitive processing, and cerebellar injury might bring about posture and movement impairment [54–56]. Previous studies have demonstrated that children with CP had smaller volumes of the cerebellar hemispheres compared to controls [57]. Chronic cerebellar stimulation applied to the superomedial cortex has been used to reduce generalized cerebral spasticity, athetoid movements, and seizures [58]. Negative correlation between the cerebellum network and GMFCS in the current study suggested that decreased functional connectivity in the cerebellum would aggravate motor dysfunction in children with CP. As part of sensorimotor-related network, SMN2 focused on the primary motor and somatosensory areas, which were vulnerable regions in CP. Disrupted sensorimotor integration has been considered as a key factor that underlies motor function in CP and other movement disorders [22, 59, 60]. In this study, the decreased connectivity near the central sulcus indicated that defective sensorimotor organization could be a relevant pathophysiological element resulting to motor dysfunction in CP.

The LFPN and SN are two functional networks referred to the task activation ensemble. Frontoparietal network is an important network in spatial attention [61], and LFPN has been mentioned to correspond well to cognition-language paradigms [31]. Studies about mortality and adverse neurological outcome in preterm infants with periventricular hemorrhagic infarction (PVHI) found that extended frontoparietal lesions were associated with the development of cerebral palsy [62, 63]. Therefore, the dysfunction within the frontoparietal network might contribute to the cognitive impairment in CP. The salience network (SN) is a large-scale paralimbic network with coactivation in response to signal for behavioral change need [64, 65]. As part of the SN, prefrontal cortex (PFC) plays an important role in goal-relevant top-down control, and a previous study has reviewed that PFC was engaged in the executive control adaption [66, 67]. In addition, the ACC has been proposed to serve in monitoring and detecting conflict, as well as error compensation, which was one useful drivers to adjust the level of executive control [66, 68]. As evidences pointed to executive function impairment in children with CP [30], the reduced connectivity within the prefrontal cortex in this study may correlate with the impaired executive function in CP, while the increased connectivity in the ACC may be the result of plasticity and compensation mechanism during development.

In the current study, common FNC alteration in DCP and SCP was revealed. Disrupted connectivity for primVN-extraVN, as well as primVN-RFPN connection, was found both in DCP and SCP. Visual perception dysfunction reflects an impairment of the capacity to process visual information, which frequently occurs in cerebral palsy and preterm children [10, 69]. As the frontoparietal network is an important network in visual-spatial attention [61], the disconnection of primVN-RFPN pathway might be one factor of visual perception impairment in CP. Moreover, decreased connectivity was exhibited in postDMN-SN and postDMN-LFPN in both DCP and SCP. The DMN, which has been studied extensively, has a set of brain regions that typically activate at rest but deactivate during performance of cognitive tasks [52, 70]. SN and LFPN are both described as task-positive networks, with activation in corresponding areas during cognitive task performance [64]. The interaction among DMN, SN, and LFPN might be related to the low-frequency toggling between the introspective and extrospective states that ensures that the individual is attentive to the unexpected or novel environmental events [71].The decreased functional connectivity between the DMN and SN/LFPN implicated the inefficient balance and regulation among these networks, which may suggest network inhibition or network unbalance as a plausible mechanism for cognitive abnormality in children with CP.

Two distinct functional disconnections were found in the DCP group compared with the SCP group, that is, cerebellum-RFPN and antDMN-SRN connections. RFPN is a network related to visual-spatial attention and somesthetic perception, while the cerebellum is the center of motor planning and motor control. Their connection, as one part of motor pathway in the brain [22], may be a vulnerable link related with the dystonic DCP. As both DMN and SRN are associated with the psychological functions of introspective and self-referential processing [52, 71, 72], the discoupling between the antDMN and SRN might relate to the disrupted cognitive self-regulation in children with DCP [73, 74]. Moreover, negative correlation between the antDMN-SRN and GMFCS, as well as positive correlations between RFPN-cerebellum, antDMN-SRN, and ADL scores, suggested that these disconnections also contribute to motor and daily activities' dysfunction in children with CP.

In this study, some children in the CP group had gray matter abnormality, and children with DCP recruited in our study tended to have more severe motion and cognitive disability compared to the SCP group. In order to investigate the effect of gray matter lesions, we took the ratio of the gray matter volume of each RSN to the TIV into consideration as covariance and reconducted the post hoc *t*-test to examine the group difference between DCP and SCP in FNC analysis. The same disconnections (cerebellum-RFPN and antDMN-SRN connection) were found in the DCP group compared with SCP. These results suggested that gray matter volume did not have a negative impact on the functional connectivity values in this study, mainly due to the strict inclusion criteria that the children with serious brain loss or lesions have been excluded.

Structure effect is one common concern in CP functional studies. Severe lesions will certainly distort the brain functional connectivity and have adverse effect in group analysis. Although children with severe brain lesions and complex clinical features were excluded in this study, potential structural effect may be inevitable. In this study, two valid measures were applied in order to weaken the effect of lesion structure to functional connectivity. On one hand, gray template mask without lesion was applied. One the other hand, ICA detects interacting networks of regions and offers the advantage to diminish the effect of abnormal signal of voxel level. During the dual-regression (temporal and spatial) back reconstruction, abnormal time courses of lesions would have slight contribution to the individual IC networks [75]. Furthermore, the FNC comparison between two CP groups, using the ratio of the gray matter volume of each network to the TIV as covariance, demonstrated slight influence of gray matter volume variation. The result suggested that the combination of participant exclusion criterion, the methodology, and reasonable template without lesions was capable to weaken the adverse impact of structural lesions during fMRI studies.

## 5. Limitations

However, there do exist some methodological issues and limitations that may affect our results. Firstly, there are a variety of brain lesions in the CP group. We chose the participants mainly according to the clinical diagnosis and MRI structure images, and there may be potential structural effect. In this study, some children with DCP have lesions in the putamen, and the lesions are excluded in the following analysis. However, the basal ganglia is an important motor-related network; the negligence of relative region in this study limited the functional connectivity analysis for the basal ganglia [17, 73]. Secondly, as most of the children with CP are accompanied by involuntary and uncontrolled movement, it is hard for them to keep still during MRI scanning for a relatively long time. Therefore, we have to scan these children under sedation state in this study. The sedation effect on the resting-state brain is inevitable. The widely recognized effects of drug-induced sedation on RSNs were the increased connectivity within motor network and visual network and the decreased connectivity in the DMN and SN [76–78]. Although our study showing inconsistent results revealed decreased connectivity in the SMN2 and increased connectivity in the ACC of the SN for CP children under sedation condition, the influence of sedation drug on functional connectivity cannot be ignored. Also, as there were only GMFCS and ADL measurement in this study, no specificity in motion and cognitive scores was applied. In the future study, more and refined related clinical scores should be adopted. Moreover, the sample size is small in this study, and future studies should include larger sample size to determine the mechanism underlying the abnormal functional connectivity found in the current study. Finally, ICA is an unsupervised method, which remains an indirect limitation when applying to identify the RSNs. The number of independent components and the reliability of IC maps are still open questions. Although we implemented ICA at different model orders and got a relatively stable result, the individual patient-specific network combined with the seed-based functional connectivity analysis and cluster analysis might provide complementary information in the comparisons.

## 6. Conclusions

This study demonstrated the functional network alteration in children with spastic and dyskinetic cerebral palsy based on resting-state fMRI. It provided a valid tool to help elucidate the abnormal functional connectivity patterns in different CP subtypes. Aberrant functional connectivity in CP groups was found, particularly within the cerebellum, SMN, LFPN, and SN networks, covering the motor- and cognitive-related networks. For FNC analysis, altered visual pathway and the covariance of DMN with other networks may be important factors of the visual perception and cognitive impairment in CP. In addition, distinct disconnections were revealed in DCP between the cerebellum and RFPN, as well as between the antDMN and SRN, which may act as an objective indicator of the clinical response in DCP. Exploring the common and distinct functional connectivity alteration is therefore beneficial for understanding the underlying mechanism of different CP subtypes and may contribute to more appropriate interventions.

## Figures and Tables

**Table 1:** Demographic and clinical data of the sample.

	DCP	SCP	HC	*P* value
*Number of participants (N*)	16	18	18	
Age (mean years ± std)	9.6 ± 5.0	8.9 ± 3.1	9.5 ± 2.2	0.818
Gender	10 M, 6 F	10 M, 8 F	11 M, 7 F	0.907
GMFCS	II: 3, III: 7, IV: 5, V: 1	I: 9, II: 5, III: 4		0.003
ADL (mean scores ± std)	38.44 ± 18	82.55 ± 3.4		<0.001
*Neuroimage finding (N*)				
White matter lesion	6	15		
Cortical gray matter abnormality	1	0		
Basal ganglia/thalamus abnormality	3	1		
Normal (*N*)	6	2		

M: male; F: female; std: standard deviation; *N*: number of participants. *P* value: comparisons for age and gender among the three groups: the variable gender was analyzed using chi-square test, while the age was analyzed using ANOVA; comparisons for GMFCS and ADL between DCP and SCP: the variable GMFCS was analyzed using chi-square test, while the ADL were analyzed using two-sample *t*-test.

**Table 2:** Significant differences of functional connectivity within four RSNs in one-way ANOVA comparison among the three groups (ANOVA, *P* < 0.05 FDR-corrected).

Networks	AAL regions	MNI coordinates			Peak *F* value	Cluster voxels
		*x*	*y*	*z*		
Cerebellum	Cerebelum_Crus1_R	27	−78	−36	31.3056	63
	Cerebelum_Crus2_R	33	−70	−38	19.3594	76
	Cerebelum_8_R	28	−65	−45	20.8600	54
	Cerebelum_Crus2_L	−15	−78	−33	19.4947	49
	Cerebelum_Crus1_L	−20	−74	−34	17.5934	43
SMN2	Postcentral_L	−54	−6	27	24.0388	25
LFPN	Parietal_Inf_L	−36	−72	39	21.8592	80
	Angular_L	−38	−66	43	16.813	37
	Occipital_Mid_L	−38	−69	41	19.2070	20
SN	Cingulum_Ant_L	−6	30	15	20.2391	47
	Frontal_Mid_R	33	36	30	17.3654	58
	Frontal_Sup_L	−18	39	36	22.8588	43
	Frontal_Mid_L	−30	34	37	14.0513.	39

MNI: Montreal Neurologic Institute; AAL: anatomical automatic labeling.

**Table 3:** Significant differences of functional connectivity within four RSNs between DCP and HC (*P* < 0.001).

Networks	AAL regions	MNI coordinates			Peak *T* value	Cluster voxels
		*x*	*y*	*z*		
Cerebellum	Cerebelum_Crus1_R	27	−78	−36	−6.9746	65
	Cerebelum_Crus2_R	22	−74	−39	−5.2359	74
	Cerebelum_8_R	21	−69	−40	−5.5828	47
	Cerebelum_Crus2_L	−15	−78	−33	−5.8267	47
	Cerebelum_Crus1_L	−20	−73	−34	−5.1369	54
SMN2	Postcentral_L	−51	−9	27	−6.5683	25
LFPN	Parietal_Inf_L	−36	−71	42	−5.0843	54
	Angular_L	−39	−66	42	−5.6904	36
SN	Frontal_Mid_R	30	36	27	−6.3314	48
	Frontal_Sup_L	−18	39	36	−7.1288	38
	Frontal_Mid_L	−30	34	38	−4.3143	39

MNI: Montreal Neurologic Institute; AAL: anatomical automatic labeling.

**Table 4:** Significant differences of functional connectivity within four RSNs between SCP and HC (*P* < 0.001).

Networks	AAL regions	MNI coordinates			Peak *T* value	Cluster voxels
		*x*	*y*	*z*		
Cerebellum	Cerebelum_Crus1_R	24	−75	−33	−5.5275	26
	Cerebelum_Crus2_R	29	−66	−41	−5.2380	27
	Cerebelum_8_R	28	−66	−44	−5.4002	42
SMN2	Postcentral_L	−51	−6	30	−5.722	25
LFPN	Parietal_Inf_L	−45	−54	51	−6.7446	78
SN	Cingulum_Ant_L	−3	33	15	6.9291	47
	Frontal_Mid_R	33	36	36	−5.3502	49
	Frontal_Sup_L	−24	39	36	−5.9748	30

MNI: Montreal Neurologic Institute; AAL: anatomical automatic labeling.

## Data Availability

The data used to support the findings of this study are available from the corresponding author upon request.

## Abbreviations

CP: Cerebral palsy
SCP: Spastic cerebral palsy
DCP: Dyskinetic cerebral palsy
RSNs: Resting-state networks
FNC: Functional network connectivity
GMFCS: Gross Motor Function Classification System
ADL: Everyday activities of daily living
SMN1: Sensorimotor network 1, included the paracentral lobule, the supplementary motor area, and pre- and postcentral gyrus
SMN2: Sensorimotor network 2, focused at the lateral primary somatosensory cortex, including pre- and postcentral gyrus areas
primVN: The primary visual network
extraVN: The extrastriate visual network.

## Supplementary Materials

Supplementary Materials The group-level spatial maps of 14 RSNs for HC, SCP, and DCP and the relationships between RSN/FNC and clinical scores in the subgroups.
