# Supplementary material for: Functional Connectivity Alterations in Children with Spastic and Dyskinetic Cerebral Palsy
Source: Neural Plast. 2018 Aug 15;2018:7058953. doi: 10.1155/2018/7058953 (PMC6114065; doi:10.1155/2018/7058953)
Supplement: Supplementary Materials — The group-level spatial maps of 14 RSNs for HC, SCP, and DCP and the relationships between RSN/FNC and clinical scores in the subgroups. [file 7058953.f1.pdf]

Supplementary Material

Captions:

Fig. S1: The group-level spatial maps of 14 RSNs for HC, SCP and DCP. SMN: sensorimotor network; DAN: dorsal attention network; antDMN: the anterior part of default mode network; postDMN: the posterior part of default mode network; SRN: the self-referential network; primVN: the primary visual network; extraVN: the extra-striate visual network; AN: the auditory network; LFPN: the left lateral frontoparietal network; RFPN: the right lateral frontoparietal network; SN: the salience network; CEN: the central executive network.

Fig. S2: Group comparison results of RSNs with different independent components when conducting ICA processing. The figures were achieved by performing one-way ANOVA on the RSN maps of the three groups, with a threshold of P<0.0005. The bar maps present the between-groups differences in the peak regions showing significant group difference in ANOVA (P<0.01). The tables showed the corresponding significant differences of functional connectivity within RSNs in one-way ANOVA comparison (ANOVA, P<0.0005).

Fig. S3: Group comparison results of FNC with different independent components when conducting ICA processing. The black line in the ANOVA result represents the altered connections among three groups (P<0.05, FDR-corrected). Then, differences of functional network connectivity between any two groups by performing hoc post test were shown (P<0.05). The blue solid line represents the connection with decreased positive FNC in CP children; and the blue dotted line represents the connection with decreased negative FNC in CP children.

Fig. S4: Relationships between RSN/FNC and clinical scores in subgroups. (A) Relationship between cerebellum network and GMFCS scores. (B) Relationship between primVN-extraVN, postDMN-SN, antDMN-SRN connection and GMFCS scores. (C) Relationship between primVN-RFPN, RFPN-cerebellum, antDMN-SRN connection and ADL scores.

Fig. S1

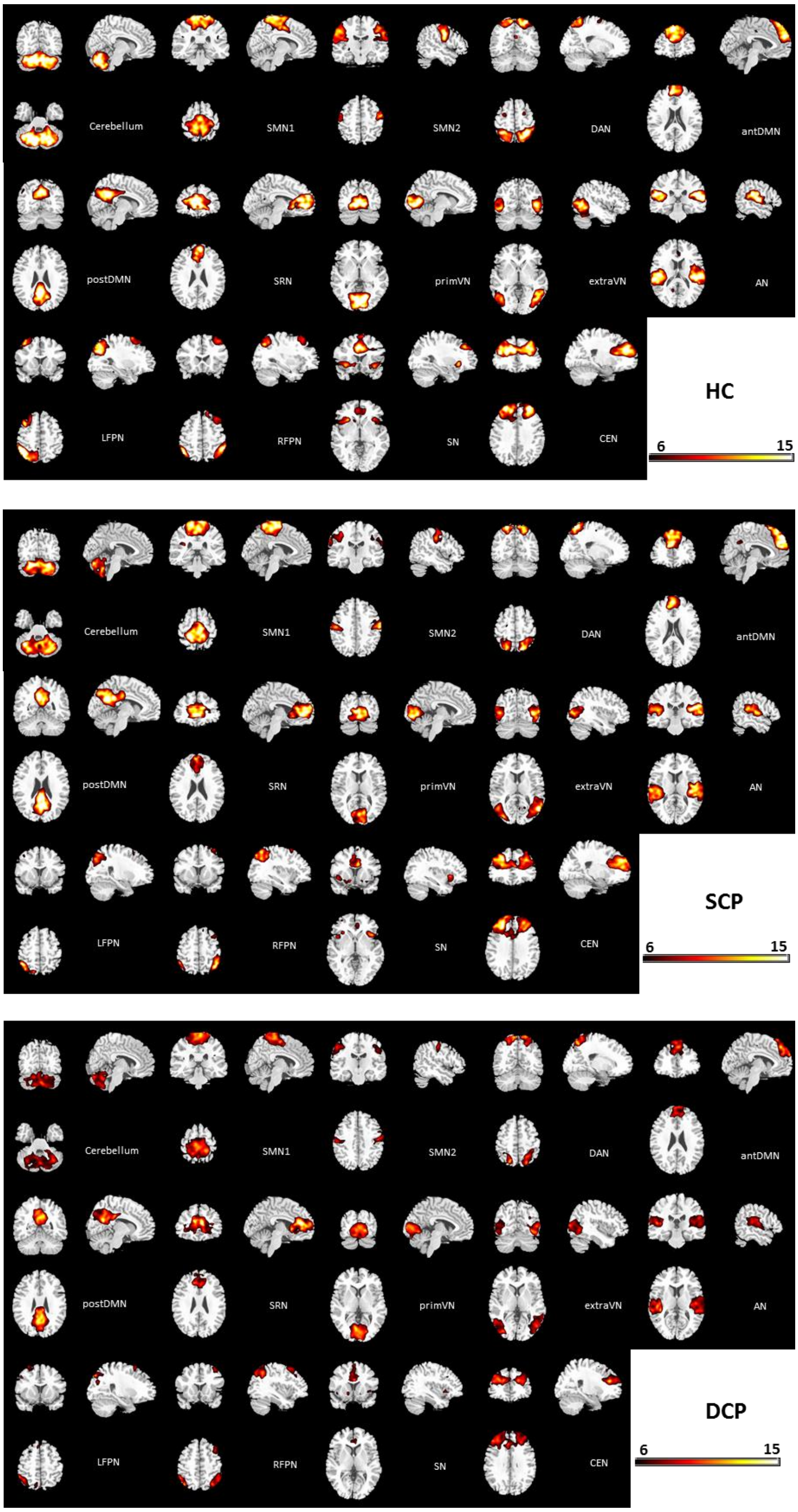
Cerebellum
SMN1
SMN2
DAN
antDMN
postDMN
SRN
primVN
extraVN
AN
LFPN
RFPN
SN
CEN
HC
6
15
Cerebellum
SMN1
SMN2
DAN
antDMN
postDMN
SRN
primVN
extraVN
AN
LFPN
RFPN
SN
CEN
SCP
6
15
Cerebellum
SMN1
SMN2
DAN
antDMN
postDMN
SRN
primVN
extraVN
AN
LFPN
RFPN
SN
CEN
DCP
6
15

Fig. S2

IC30:

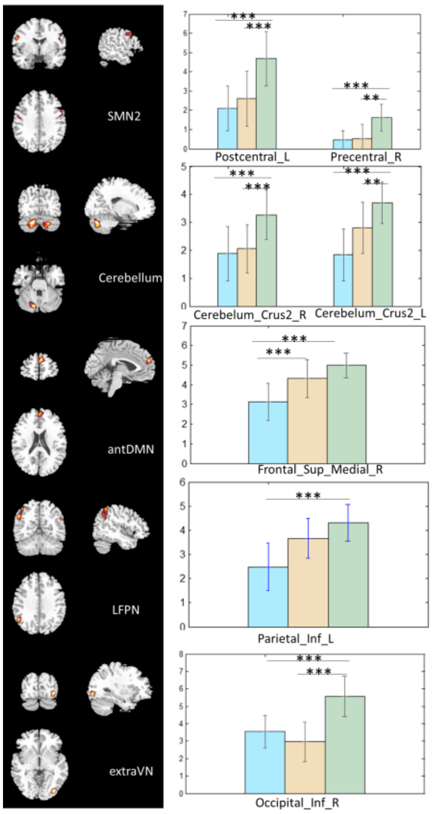

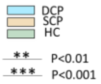

		MNI coordinates				
Networks	AAL Regions	X	Y	Z	Peak T-value	Cluster voxels
SMN2	Postcentral_L	-54	-6	24	20.2281	21
	Precentral_R	54	9	33	15.2451	20
Cerebellum	Cerebelum_8_R	30	-66	-45	22.1961	56
	Cerebelum_Crus2_R	30	-65	-42	22.7442	44
	Cerebelum_Crus1_R	16	-73	-33	19.6169	22
	Cerebelum_Crus2_L	-15	-78	-33	26.6077	84
	Cerebelum_Crus1_L	-18	-75	-33	21.9306	55
aDMN	Frontal_Sup_Medial_R	6	54	27	20.3701	45
LFPN	Angular_L	-51	-60	36	16.7704	43
	Parietal_Inf_L	-39	-62	55	19.2455	49
extraVN	Occipital_Inf_R	36	-84	-6	26.9826	40

IC35:

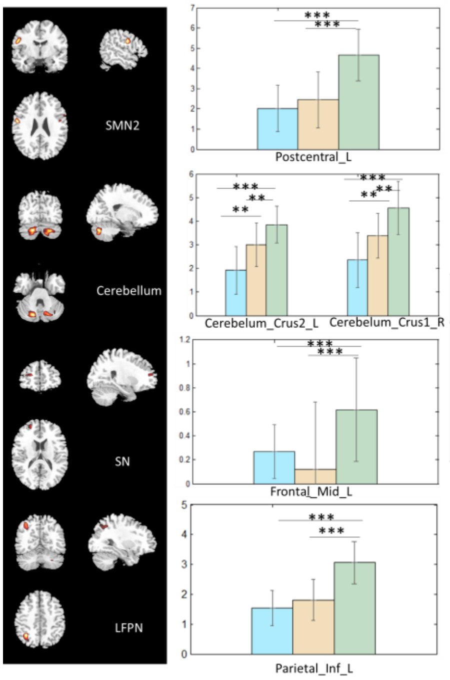

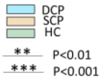

		MNI coordinates				
Networks	AAL Regions	X	Y	Z	Peak T-value	Cluster voxels
SMN2	Postcentral_L	-54	-6	24	22.7513	27
	Precentral_R	57	6	36	17.614	32
Cerebellum	Cerebelum_Crus2_L	-15	-78	-33	22.0059	60
	Cerebelum_Crus1_L	-19	-73	-33	14.792	39
	Cerebelum_Crus1_R	27	-78	-36	26.1875	37
	Cerebelum_Crus2_R	21	-76	-40	16.6602	58
SN	Frontal_Mid_L	-21	48	12	13.8879	23
LFPN	Parietal_Inf_L	-33	-57	45	28.9658	60

IC40:

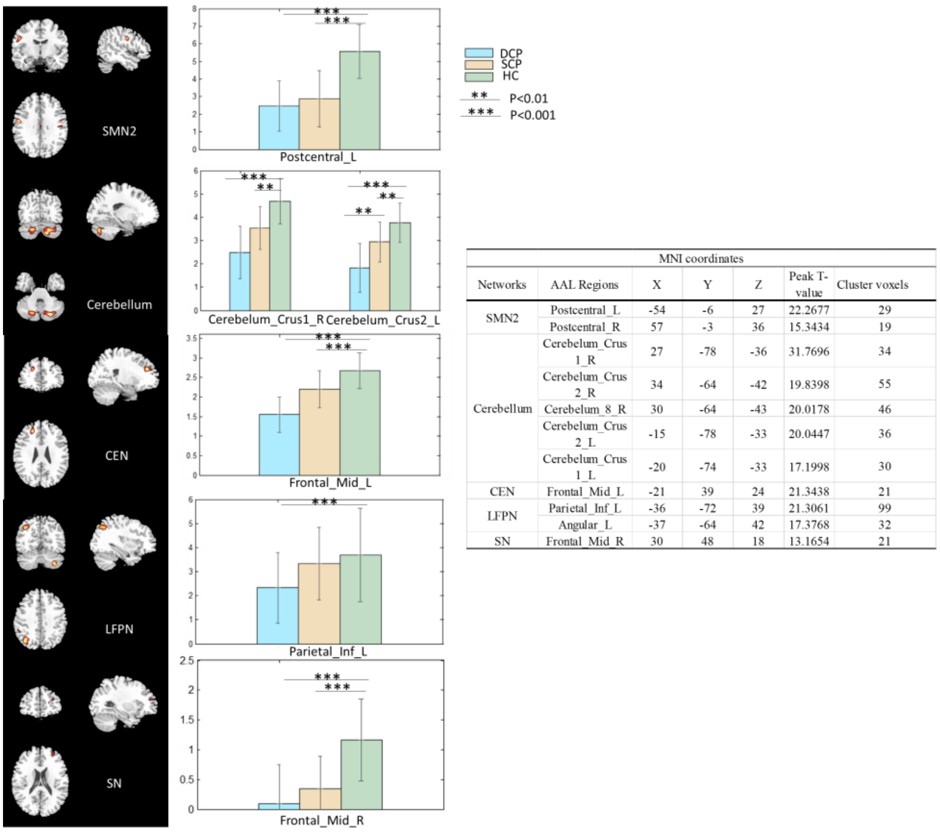

		MNI coordinates				
Networks	AAL Regions	X	Y	Z	Peak T-value	Cluster voxels
SMN2	Postcentral_L	-54	-6	27	22.2677	29
	Postcentral_R	57	-3	36	15.3434	19
Cerebellum	Cerebelum_Crus 1_R	27	-78	-36	31.7696	34
	Cerebelum_Crus 2_R	34	-64	-42	19.8398	55
	Cerebelum_8_R	30	-64	-43	20.0178	46
	Cerebelum_Crus 2_L	-15	-78	-33	20.0447	36
	Cerebelum_Crus 1_L	-20	-74	-33	17.1998	30
CEN	Frontal_Mid_L	-21	39	24	21.3438	21
LFPN	Parietal_Inf_L	-36	-72	39	21.3061	99
	Angular_L	-37	-64	42	17.3768	32
SN	Frontal_Mid_R	30	48	18	13.1654	21

IC45:

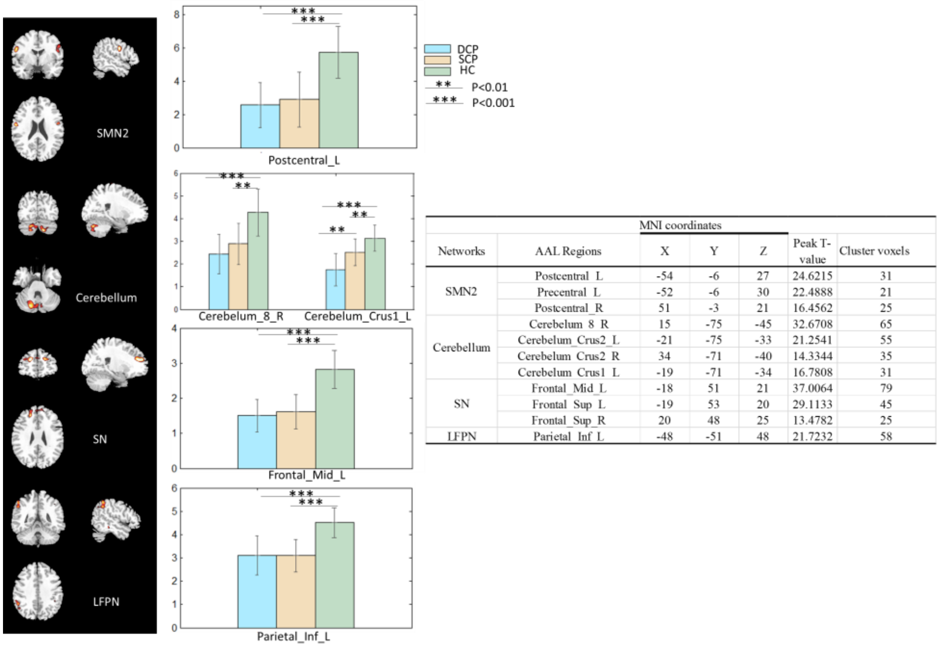

		MNI coordinates				
Networks	AAL Regions	X	Y	Z	Peak T-value	Cluster voxels
SMN2	Postcentral_L	-54	-6	27	24.6215	31
	Precentral_L	-52	-6	30	22.4888	21
	Postcentral_R	51	-3	21	16.4562	25
Cerebellum	Cerebelum_8_R	15	-75	-45	32.6708	65
	Cerebelum_Crus2_L	-21	-75	-33	21.2541	55
	Cerebelum_Crus2_R	34	-71	-40	14.3344	35
	Cerebelum_Crus1_L	-19	-71	-34	16.7808	31
SN	Frontal_Mid_L	-18	51	21	37.0064	79
	Frontal_Sup_L	-19	53	20	29.1133	45
	Frontal_Sup_R	20	48	25	13.4782	25
LFPN	Parietal_Inf_L	-48	-51	48	21.7232	58

IC50:

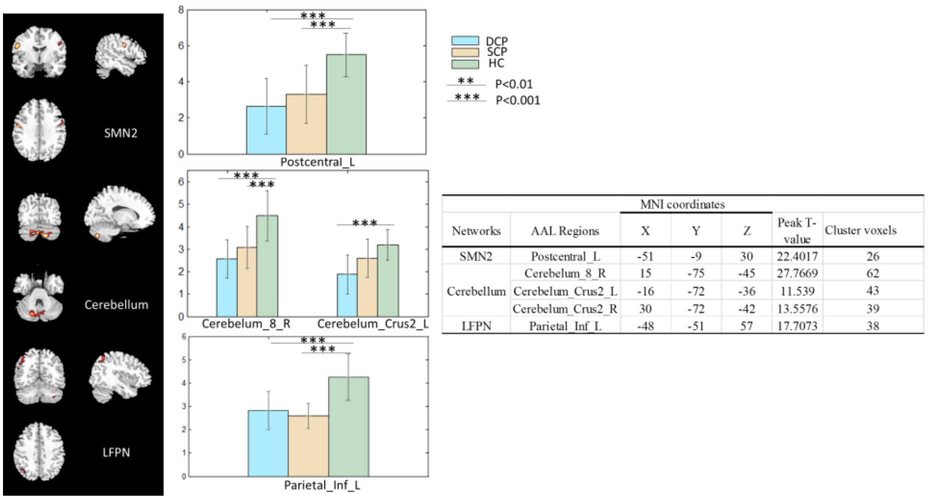

		MNI coordinates				
Networks	AAL Regions	X	Y	Z	Peak T-value	Cluster voxels
SMN2	Postcentral_L	-51	-9	30	22.4017	26
Cerebellum	Cerebelum_8_R	15	-75	-45	27.7669	62
	Cerebelum_Crus2_L	-16	-72	-36	11.539	43
	Cerebelum_Crus2_R	30	-72	-42	13.5576	39
LFPN	Parietal_Inf_L	-48	-51	57	17.7073	38

IC55:

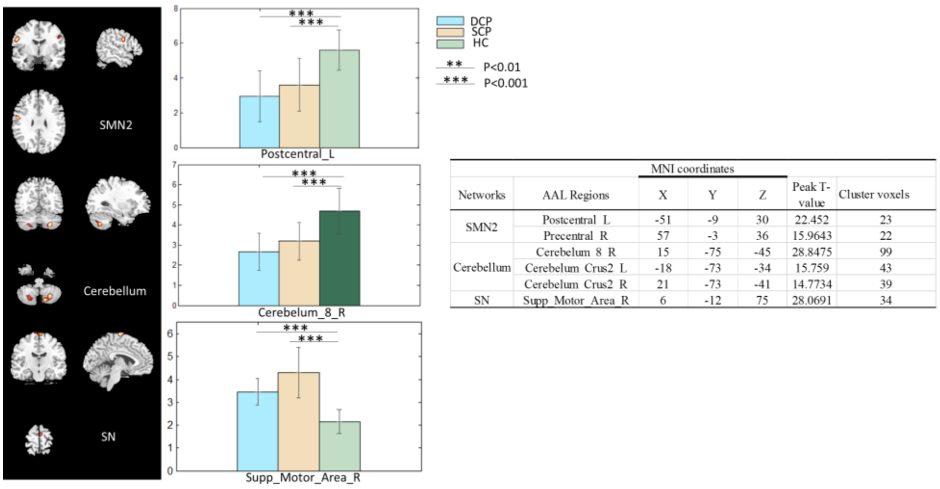

		MNI coordinates				
Networks	AAL Regions	X	Y	Z	Peak T-value	Cluster voxels
SMN2	Postcentral_L	-51	-9	30	22.452	23
	Precentral_R	57	-3	36	15.9643	22
Cerebellum	Cerebelum_8_R	15	-75	-45	28.8475	99
	Cerebelum_Crus2_L	-18	-73	-34	15.759	43
	Cerebelum_Crus2_R	21	-73	-41	14.7734	39
SN	Supp_Motor_Area_R	6	-12	75	28.0691	34

IC60:

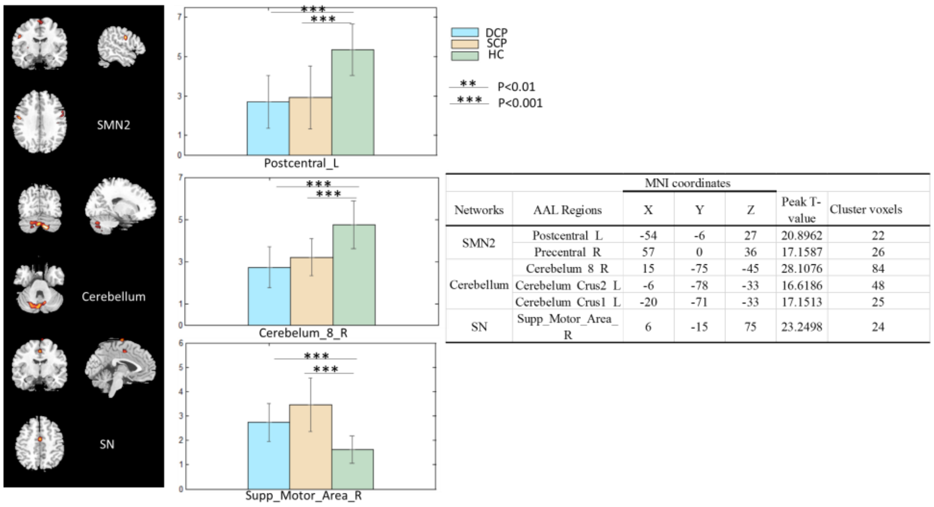

		MNI coordinates				
Networks	AAL Regions	X	Y	Z	Peak T-value	Cluster voxels
SMN2	Postcentral_L	-54	-6	27	20.8962	22
	Precentral_R	57	0	36	17.1587	26
Cerebellum	Cerebelum_8_R	15	-75	-45	28.1076	84
	Cerebelum_Crus2_L	-6	-78	-33	16.6186	48
	Cerebelum_Crus1_L	-20	-71	-33	17.1513	25
SN	Supp_Motor_Area_R	6	-15	75	23.2498	24

Fig. S3

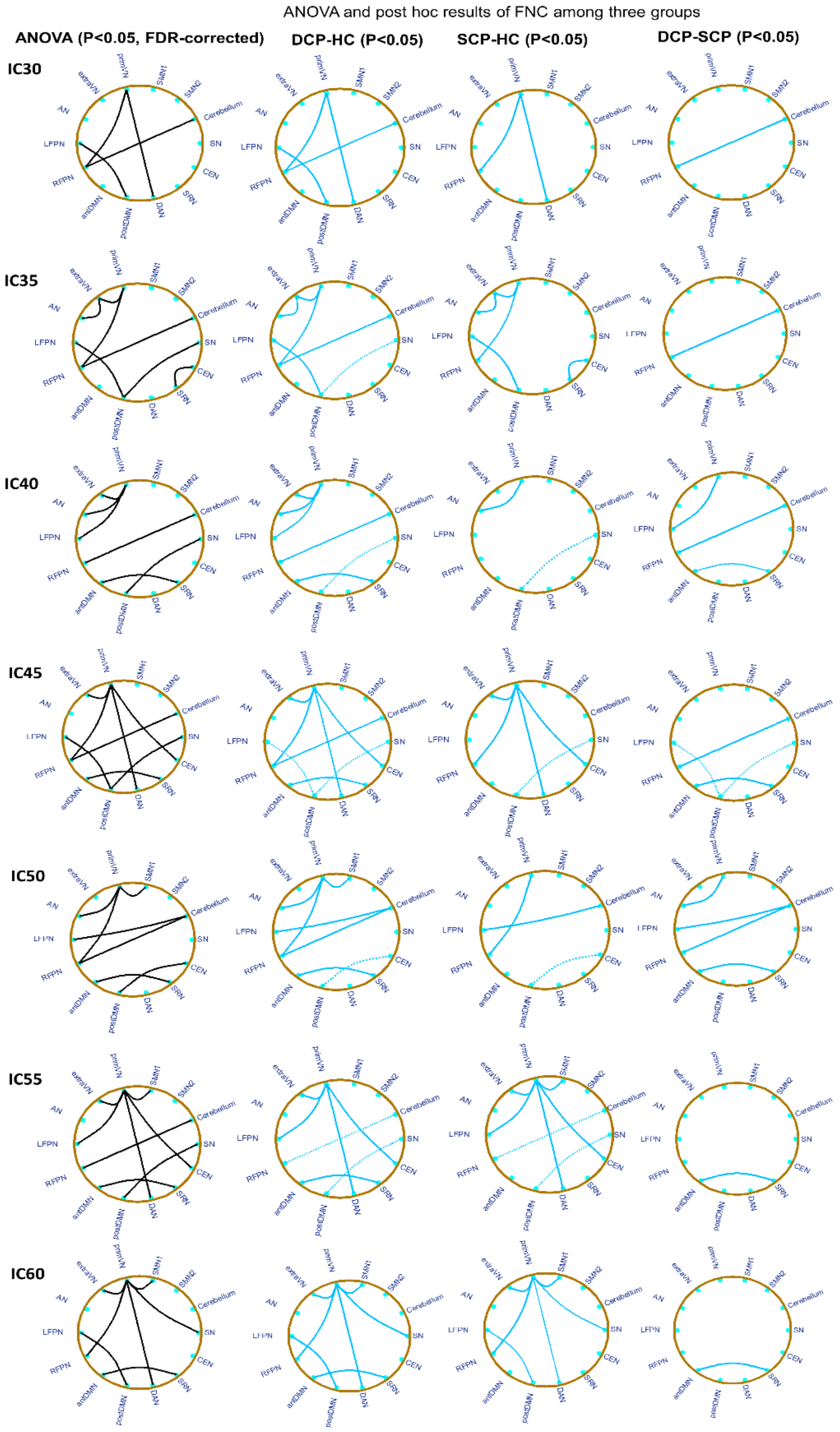
ANOVA and post hoc results of FNC among three groups
ANOVA (P<0.05, FDR-corrected)
DCP-HC (P<0.05)
SCP-HC (P<0.05)
DCP-SCP (P<0.05)
IC30
IC35
IC40
IC45
IC50
IC55
IC60
primVN
SMN1
SMN2
Cerebellum
SN
CEN
SRN
DAN
postDMN
antDMN
RFPN
LFPN
AN
extraVN

Fig. S4

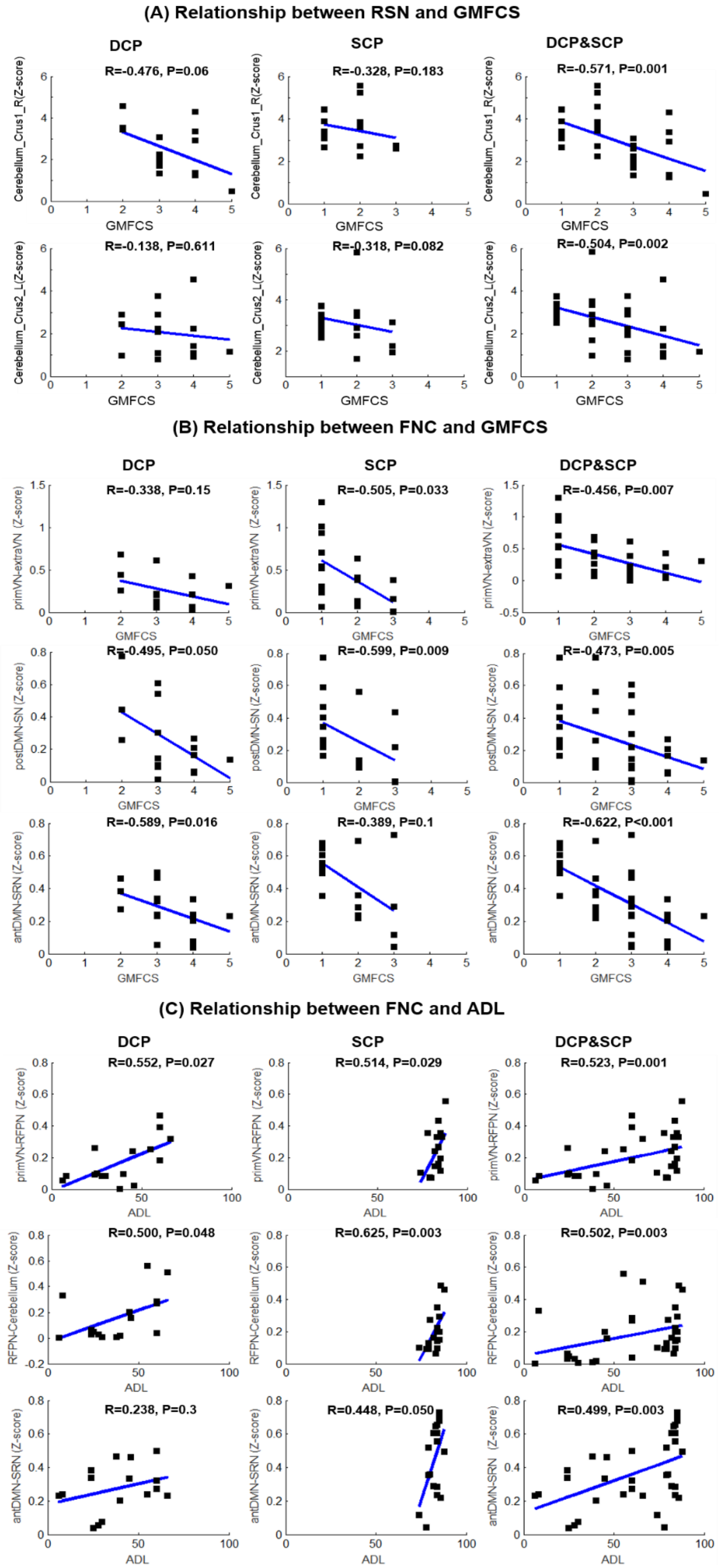
(A) Relationship between RSN and GMFCS
DCP
SCP
DCP&SCP
R=-0.476, P=0.06
R=-0.328, P=0.183
R=-0.571, P=0.001
Cerebellum_Crus1_R(Z-score)
GMFCS
R=-0.138, P=0.611
R=-0.318, P=0.082
R=-0.504, P=0.002
Cerebellum_Crus2_L(Z-score)
(B) Relationship between FNC and GMFCS
R=-0.338, P=0.15
R=-0.505, P=0.033
R=-0.456, P=0.007
primVN-extraVN (Z-score)
R=-0.495, P=0.050
R=-0.599, P=0.009
R=-0.473, P=0.005
postDMN-SN (Z-score)
R=-0.589, P=0.016
R=-0.389, P=0.1
R=-0.622, P<0.001
antDMN-SRN (Z-score)
(C) Relationship between FNC and ADL
R=0.552, P=0.027
R=0.514, P=0.029
R=0.523, P=0.001
primVN-RFPN (Z-score)
ADL
R=0.500, P=0.048
R=0.625, P=0.003
R=0.502, P=0.003
RFPN-Cerebellum (Z-score)
R=0.238, P=0.3
R=0.448, P=0.050
R=0.499, P=0.003
antDMN-SRN (Z-score)
